# Temporal Evolution of Interparticle Potentials of PMMA Colloids in CHB/Decalin

**DOI:** 10.1021/acs.langmuir.4c00905

**Published:** 2024-07-26

**Authors:** Marcel Rudolf, Andreas Zumbusch

**Affiliations:** Department of Chemistry, Universität Konstanz, D-78457 Konstanz, Germany

## Abstract

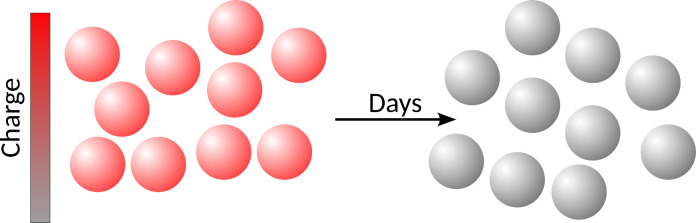

Colloidal dispersions composed of polymethylmetacrylate particles dispersed in a mixture of cyclohexyl bromide and decalin find widespread use as model systems in optical microscopy experiments. While the system allows simultaneous density and refractive index matching, preparing particles with hard potentials remains challenging, and strong variations in the physical parameters of samples prepared in the same manner are commonly observed. Here, we present data on the measurement of forces between individual pairs of particles in highly diluted dispersions over the course of tens of days using the blinking optical tweezers method. Our results show that the variations in the particle properties are indeed caused by a temporal evolution of the particles’ charging. Additional measurements of the influence of the addition of tetrabutylammonium bromide (TBAB) to the dispersions show that already small concentrations of added TBAB salt drastically decrease the electrostatic forces between colloidal particles. However, small, non-negligible contact potentials remain even at the highest TBAB concentrations added.

## Introduction

Apart from their importance in nature and technology, colloidal particles are attractive models for atomic and molecular systems.^1^ With typical diameters in the micrometer range, they are easily visualized e.g. using confocal fluorescence microscopy.^2,3^ In such optical experiments, colloidal particles can be considered as ’big atoms’ that are detectable on an individual particle basis.^4^ The tracking of thousands of individual particles gives information that is not accessible in atomic or molecular samples.^5^ A prototypical example for a phenomenon that can be studied in this manner is the glass transition.^6^

To use colloids as models in optical microscopy experiments, a number of prerequisites has to be fulfilled. In most cases one needs to simultaneously match the density and the refractive index of the colloidal particles to the dispersion medium used: Density matching has the advantage of reducing the movement of particles due to sedimentation during measurements. Refractive index matching of particles and fluid, by contrast, reduces scattering, thus allowing imaging of the particles deep in the sample, and reduces van der Waals forces between particles. To date, a few combinations of particles and dispersion media are known for which density and refractive index can be matched at the same time.^7−10^ A widely used combination consists of sterically stabilized polymethylmetacrylate (PMMA) colloids in a solution of cyclohexyl bromide (CHB) and *cis*-decalin.^11−17^ Apart from the two physical properties just discussed, a lot of effort is often taken to prepare particles with hard sphere potentials, i.e. an infinitely high potential for distances smaller than the particle diameter and zero potential everywhere else. Particles of this type are attractive due to their physical simplicity, since for suspensions of particles with perfectly hard potentials, volume fraction is the only parameter controlling structure and dynamics in dense suspensions and no interparticle forces have to be considered in their theoretical description. However, charging often leads to soft particle potentials.^18^ In addition, strong variations of parameters like Debye lengths, particle ζ potentials, and particle charges are commonly observed even for samples prepared in the same manner.^19^ Since the electrostatic properties of dispersed colloids are known to be very susceptible to impurities such as ions or glue residues introduced by the sample chamber preparation,^20^ one usually assumes imperfections in the preparation as causes for the observed variations.

Particles with potentials closely resembling hard spheres can be prepared when the two main contributions to the potential are screened. These contributions are electrostatic and van der Waals forces. To overcome the attractive forces arising from van der Waals forces, frequently a steric stabilizer consisting of poly(12-hydroxystearic acid) (PHSA) is covalently attached to the particles. While the chains of the stabilizer create a certain softness in the potential, surface force measurements proved that the influence of the stabilizer on the potential is short-range and not measurable for distances greater 20 nm away from the particles’ surfaces.^21^ Thus, especially for spheres with diameters in the μm range, the stabilizer has only little effect on the hardness of the potential. The main deviation from hard potentials therefore arises from electrostatic forces. Several approaches have been used to gain information on electrostatic particle potentials. Indirect information about the potential between pairs of particles has been obtained in different manners: by comparing the radial distribution function of a particle ensemble with results of its theoretical description or simulation results,^22−26^ by measuring the conductivity and mobility with electrophoresis,^11,19,22,24,27,28^ and by examination of the crystallization behavior of dispersions as a function of volume fraction.^11,23,29,30^ Direct measurements of interparticle forces are possible using optical tweezers to trap particles while monitoring their interaction via distance measurements. Variations of this approach have been used to study interparticle potentials in a number of different systems.^10,20,31−36^

Here, we report quantitative measurements of interparticle forces based on a method known as blinking optical tweezers, first established by Crocker and Grier^37^ and later modified by Sainis and co-workers.^31^ With such an experiment, we deduce interparticle potentials for individual PMMA particle pairs in mixtures of CHB and decalin. To trap PMMA particles in the index matching solvent, we use an approach that consists in using core/shell particles with a refractive index matching shell and a higher refractive index core material. The cores can then be trapped by optical tweezers.^10^ In our case, PMMA colloids containing a polystyrene (PS) core are employed.^38^ Due to the refractive index mismatch of the core, no labeling of the particles is necessary and their positions can be tracked with bright-field microscopy. Since the chosen core to shell volume ratio is 1:46, the particles used can be assumed to very closely mimic the behavior of pure PMMA colloids. With this system, we investigate the temporal changes of forces between pairs of colloids over the course of tens of days. While all particles initially are significantly charged, we find that the charging decreases by a factor of 3 within 5 days. In order to minimize the effect of charging, organic salts such as tetrabutylammonium chloride (TBAC) or bromide (TBAB) are commonly added to the dispersions.^15,17,39−41^ This strategy is also employed for other systems similar to PMMA in CHB/decalin.^42,43^ We therefore also investigated, how the addition of TBAB to a dispersion of PMMA particles in CHB/decalin affects the interparticle forces and found that already small amounts of TBAB lead to a significant hardening of the particle potentials. Temporal changes of the interparticle forces then become negligible. Yet, even at the highest TBAB concentrations tested, the potentials retained a non-negligible softness.

## Results and Discussion

### Particle Synthesis

**Figure 1:**
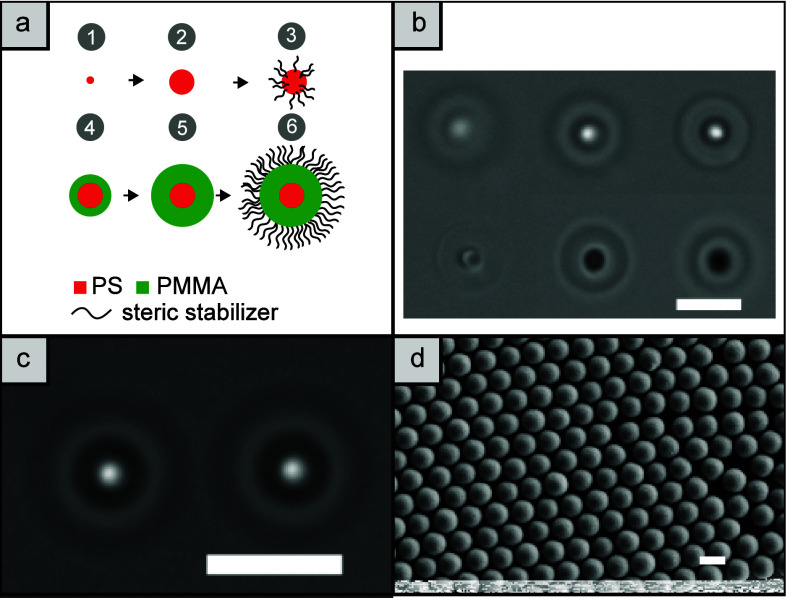
(a) Particle synthesis (1) PS seeds synthesis in aqueous solution. (2) First and second PS shell growth. (3) Adhesion of a steric stabilizer and transfer to *n*-hexane/dodecane. (4, 5) PMMA shell growth. (6) Smoothing and covalent bonding of the steric stabilizer. (b) Bright field images of a particle when moved along the *z*-axis with the optical tweezers (from upper right to lower left). While the PS core remains visible, the PMMA shell contrast vanishes when in focus. (c) Experimental bright field image. (d) SEM image of the core/shell particles. Scale bars: 5 μm.

The protocol for the synthesis of the PS/PMMA core/shell particles shown in Figure 1 was adapted from Klein et al.^38^ Following this procedure, first PS seed particles with a diameter of 190 nm were synthesized. Their diameter was increased to 600 nm by growing an additional PS layer in an emulsion polymerization step. To this end, 35 ml doubly distilled water (Carl Roth) and 0.6 ml of the cores suspended in water (solid content 3.4%) were heated to 73 °C under nitrogen in a 100 ml Schlenk flask. Meanwhile, 73 mg of potassium peroxodisulfate (Sigma Life Science) were mixed with 10 ml doubly distilled water and a monomer mixture consisting of 10 g of distilled styrene (Merck) and 0.4 g of 1,3-diisopropenylbenzene (DIPB, Tokyo Chemical Industries) was prepared. One ml of the K_2_S_2_O_8_ mixture was given to the particles. Ten min later the nitrogen flow was stopped and 1.6 ml of the monomer mixture were added with a rate of 0.8 ml/h using a syringe pump. The particles were stirred for another 2 h, then cooled down to room temperature, and filtered with glass wool. The same seeded emulsion polymerization step was repeated with 4 ml of the synthesized particle solution and 32 ml of doubly distilled water to create bigger PS cores with a diameter of 1.5 μm. These were then transferred to *n*-hexane/dodecane as described by Klein et al.^38^ First, the particles were centrifuged and the supernatant was replaced with acetone. We found it necessary to extend the duration for which the particles were kept in acetone to at least 12 h. Then the particles were transferred to 19.5 g of petroleum ether (PE) (boiling point 40–60 °C, Sigma-Aldrich). Thirty drops of the steric stabilizer poly(12-hydroxystearic acid) (PHSA) grafted onto a backbone of PMMA (PHSA–*g*–PMMA)^44^ were added. After 2 min of sonication, the particles were washed three times with PE and sonication in between, before transfer to a mixture of *n*-hexane/dodecane (2:1 (wt %:wt %)) (*n*-hexane: for HPLC, VWR Chemicals). The particles were again centrifuged and filled up with 1.23 g of the *n*-hexane/dodecane solution.

With the PS cores as seed, a seeded dispersion polymerization step was used to grow a first PMMA shell resulting in particles with a diameter of 2.8 μm. For this purpose, a monomer mixture consisting of 21.3 ml methyl methacrylat (Sigma-Aldrich), 0.39 ml methacrylic acid (Sigma-Aldrich), 2.5 mL stabilizer, and 20.8 g of *n*-hexane/dodecane mixture was prepared. In a 25 ml Schlenk tube, 39 mg of azo-bis-isobutyronitrile (Sigma-Aldrich) and the PS seed particles in *n*-hexane/dodecane were stirred with 250 rpm with a 1 cm magnetic stirrer bar. 8 μl of octyl mercaptan (Sigma-Aldrich) were added and the particles were heated to 95 °C. 4.8 g of the monomer mixture were added at a rate of 3 ml/h using a syringe pump. After waiting for 2 h and cooling down to room temperature, the particles were filtered, and washed with PE. After the last centrifugation, the supernatant was removed and the particles were filled up with 2 ml PE. 0.3 ml of the particles were surface smoothed with a mixture of 9.7 g of *cis*/*trans*-decalin (>98%, Carl Roth) and 1.5 g of acetone and 2 drops of stabilizer for 40 min.^45^ It was important to thoroughly mix the smoothing solution before adding the particles. After quenching with 25 mL decalin, the particles were then transferred to pure decalin. 0.29 g (solid weight) of these particles were used again as seed particles for another seeded dispersion polymerization. This resulted in 1.6 g of particles. The particles were again smoothed with a mixture of 135 g of decalin and 21 g of acetone for 1.5 h before transfer to pure decalin. Finally, the particles were sterically stabilized by locking (PHSA–*g*–PMMA) covalently onto the particles’ surface. During smoothing, some particles coalesced. These were removed by sedimentation. The final particles had a core diameter of 1.42 ± 0.08 μm (determined with SEM, 5.4% polydispersity) and a shell diameter of 5.07 ± 0.12 μm (determined with SEM, 2.5% polydispersity). Since the particles are know to swell in decalin,^26^ we kept the particles for at least 7 days in decalin before further usage. After swelling, the particles had a diameter of 5.8 μm.

### Sample Preparation

The solvent for the particles was a (85 wt %/15 wt %) mixture of CHB (>98%, Tokyo Chemical Industries) and decalin. We only used CHB from freshly opened bottles, as the ion concentration in CHB is changing over time due to dissociation.^20,36,46,47^ To avoid contact between particles, the particle concentration was kept smaller than 10 per μl, equivalent to a volume fraction of ϕ ≈ 8 · 10^–7^. In some experiments, tetrabutylammonium bromide (TBAB, Sigma-Aldrich) was added to quantify the screening effect of this salt. In these cases, first a 368 μM solution of TBAB dissolved in CHB was prepared under constant stirring in nitrogen atmosphere over a minimum of 3 days. To this end, 100.74 g of CHB from a freshly opened bottle were directly filled into a 250 ml Schlenk flask using N_2_ as inert gas. 9 mg of TBAB were then added under constant N_2_ flow. The dispersion was stirred for 3 days under N_2_ with a magnetic stir bar. To avoid photodecomposition of CHB, the Schlenk flask was shielded with aluminum foil. The resulting solution was used as stock solution from which the mixtures with fresh CHB and decalin and colloids in decalin were prepared. When necessary, this solution was diluted with CHB. Then the CHB/TBAB solution was mixed with decalin containing the particles.

The sample chamber consisted of a glass slide (75 × 25 × 3 mm^3^), with a centered round pit (diameter 8 mm, depth 0.5 mm) on the bottom side. From the other side, an additional small hole (diameter 2.8 mm, depth 2.5 mm) was cut. Before usage, these glass slides and glass coverslips (18 × 18 × 0.17 mm^3^, Marienfeld) were washed for an hour in an ultrasonic bath set to 45 °C first with doubly distilled water and then for another hour in ethanol (for spectroscopy, Uvasol Supelco) before drying in a nitrogen flow. Care was taken to touch the glasses only with clean tweezers. The bottom whole was covered with a coverslip and the edges were sealed with an epoxy resin (UHU Plus Sofortfest). As the resin is known to influence the interparticle potential when not properly hardened,^20^ we waited at least 12 h before filling in the sample liquid. After the liquid was inserted with a glass pipet, another glass coverslip was put on the top hole and sealed with epoxy resin. To avoid contact of the soft resin with the sample liquid, we waited for another 12 h before moving the sample chamber.

### Experimental Setup and Raw Data Processing

The experimental setup consists of a diode laser (P = 250 mW, λ = 785 nm, FPL785S250, Thorlabs). The laser beam is directed over a spatial light modulator (SLM, X10468, Hamamatsu) and coupled via relay optics into a microscope (DMI 6000B, Leica). Phase masks were sent to the SLM to form two optical traps in the sample. Interparticle forces were deduced with a well-established method.^31^ In brief, two particles were trapped and released periodically at initial distances Δ*R* between the particle centers. The laserdiode was switched on and off by a program every 500 ms. The off-times were 100 ms. During the time the traps were switched off, images were taken with an exposure time of 250 μs at a rate of 1000 fps using a CMOS camera (mvBlueFOX3–2 2004 C, Matrix Vision). Pixel sizes in the image plane were 115 nm x 115 nm. The free diffusing particles were recorded for 100 frames and from the resulting images, two-dimensional trajectories *r*_1_ (*t*) and *r*_2_ (*t*) were generated. A single measurement then consisted of a video sequence composed of 100 individual images recorded with a frame rate of 1000 fps. This was repeated roughly 3 · 10^4^ times for a pair of particles.

**Figure 2:**
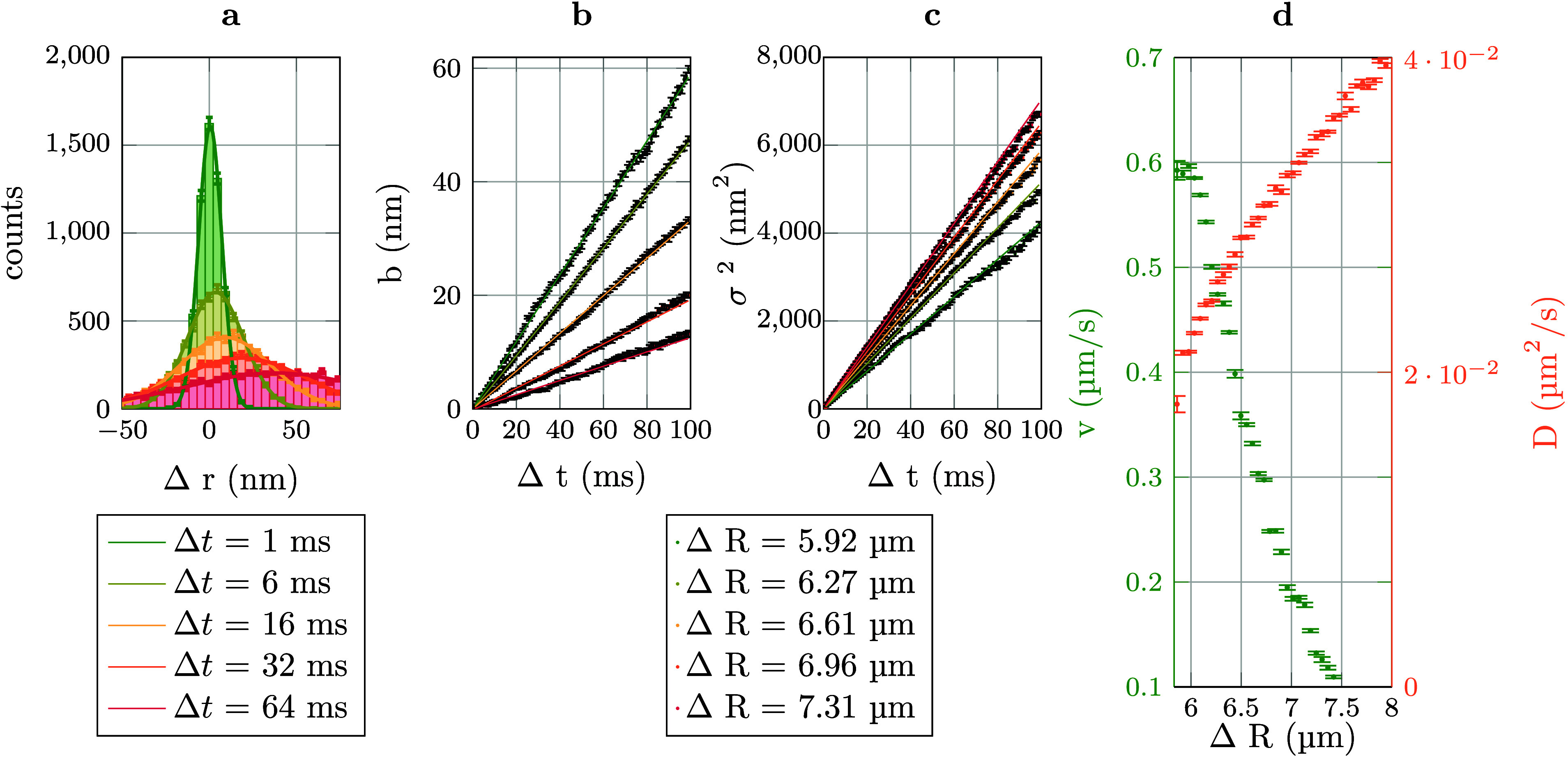
Raw data processing. The depicted data were taken from a sample without added TBAB. (a) Histogram of Δ*r*, the change in particle separation after an interval Δ*t*. For this histogram, the initial distance for all particle pairs was ΔR = 5.95 μm. The histogram well fitted by a Gaussian distribution. (b,c) Parameters *b* and σ^2^ of the Gaussian fits from (a). (d) The parameters *v* and *D*, that are derived from the parameters *b* and σ. From *v* and *D*, the force *F* is calculated. Error bars shown are 1 σ confidence intervals.

To outline the data processing, exemplary data from samples without added TBAB are depicted in Figure 2. Figure 2a shows the measured distance distribution of Δ*r* = (*r*_1_ (*t*) – *r*_2_ (*t*)) – (*r*_1_ (*t*_0_) – *r*_2_ (*t*_0_)) for several time intervals Δ*t* = *t* – *t*_0_. Here, 1 and 2 refer to particle 1 and particle 2, respectively. These curves are fitted with a Gaussian distribution function *p*(Δ*r*) = *A* · exp((Δ*r* – *b*)^2^/(2σ^2^)). The parameters *b* and σ^2^ are linearly dependent on Δ*t* (Figure 2b and Figure 2c). A linear fit yields *v* = (d *b*)/(d Δ*t*) and *D* = (d σ^2^)/(2d Δ*t*) (Figure 2d). With these parameters the forces are calculated using^31^*F* = (*k*_*B*_*T* · *v*)/*D*

The particles were slightly defocused during the measurements. This facilitates the fitting of the observed two-dimensional intensity distribution using Gaussian functions (see Figure 1 b and c). In this manner, particle positions could be determined with subpixel precision. As the distance between measurement spot and glass surface might have an influence on the measured potentials,^22^ all data were acquired at depths 100 μm above the lower glass coverslip surface.

### Results

#### Temporal Evolution of Interparticle Forces

**Figure 3:**
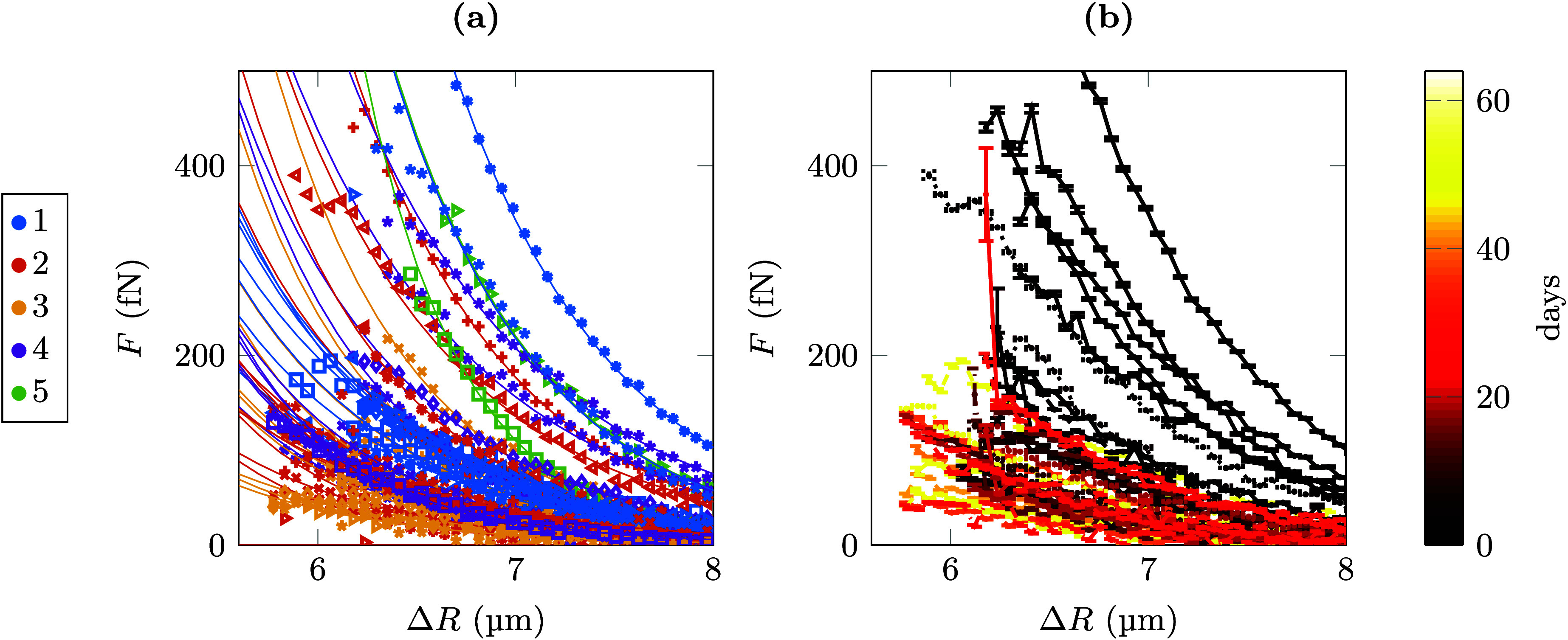
Interparticle forces determined from PMMA particles in CHB/decalin without added TBAB. (a) All data were recorded from different sample chambers plotted together. Color encodes for the different sample chambers used. With each chamber, several measurements were performed. (b) The same data as in (a) but now color coded for time after sample preparation. Error bars shown are 1 σ confidence intervals.

In a first series of experiments, we investigated samples of PMMA particles in CHB/decalin without added TBAB. Figure 3a shows all data recorded from different sample chambers plotted together. Since the force data are expected to be highly susceptible to impurities introduced during sample preparation, especially ions and glue residues in the sample fluid, we wondered whether the strong variations in the determined force curves reflected the purity of the samples. This reasoning was motivated by previous report of similar variations in parameters like Debye lengths and effective charges for different samples despite their careful preparation.^19,26^ Sorting the same data as a function of time after sample preparation, however, shows that the observed variations are not caused by irregularities in the sample preparation, but are rather much due to a change of the samples as a function of time after preparation (cf. Figure 3b).

**Figure 4:**
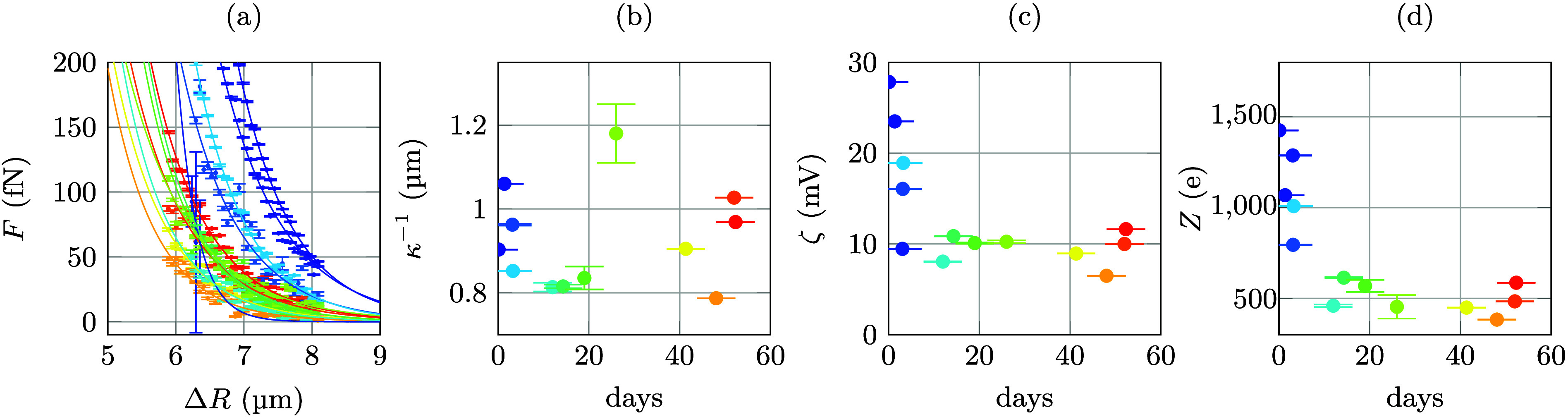
Temporal evolution of the interparticle forces as a function of time after sample preparation. (a) Force curves from one sample cell without added TBAB. From the data, Debye lengths κ^–1^ (b), surface potentials ζ (c), and surface charges Z (d) as a function of days after the preparation of the sample cell are derived. Error bars shown are 1 σ confidence intervals.

This observation prompted us to analyze the data for a long series of experiments on one sample chamber in greater detail (Figure 4). All the force measured in our experiments are well described by a screened Coulomb force^31^
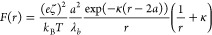
1where *e* is the elementary charge, ζ is the surface potential, *a* is the particle radius, λ_*B*_ = *e*^2^/(4*πϵ ϵ*_0_*k*_*B*_*T*)) is the Bjerrum length, and κ^–1^ is the Debye screening length. The solid lines shown in the force data of Figure 4 are fits with eq 1, where κ and ζ are fitting parameters. To account for particle polydispersity, the particle radius *a*, by contrast, was determined from bright-field microscopy. This was done by focusing the particles such that their outer rims became slightly visible. Knowing the Debye length κ and the surface potential ζ, an apparent surface charge can be calculated^22^
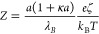
2

Our data show that the interaction between the colloids stabilizes roughly 5 days after sample preparation. The values for the determined Debye lengths are scattered between 0.8 and 1.2 μm, but show no trend in their temporal evolution. This means that the ion concentration in the dispersion fluid hardly changes with time. ζ potentials and, as a result also the apparent surface charges Z of the colloids, by contrast, clearly show a rapid decay immediately after sample preparation, before settling at constant values of approximately one-third of their starting values after 5 days. The number of surface charges Z changes from a maximum of roughly 1400 to a plateau value of 500 elementary charges *e* corresponding to a change from 17 *e* μm^–2^ to 6 *e* μm^–2^. The initial value compares well to recent data reported for a similar system (the particles in this case were labeled with a cationic dye) where Z = 15 *e* μm^–2^ to Z = 30 *e* μm^–2^ was found.^26^ In this publication, the authors report a strong dependence of Z on the volume fraction ϕ in a range of 0.1 < ϕ < 0.49. While our experiments were done in highly diluted dispersions with ϕ ≈ 8 · 10^–7^ one might speculate that contacts between particles and between particles and the chamber surfaces contribute to the discharging of the particles.

It has been postulated that the charging of colloidal particles in CHB could be due to its dissociation and slightly preferential adsorption of protons.^19,20^ Our method does not allow us to draw conclusions about the sign of the charges on the colloids, as ζ appears quadratically in eq 1. However, we can infer from the stable observed Debye lengths that in the samples investigated, no significant decomposition of CHB took place after their preparation. This does not exclude the possibility that a certain number of ions present in the CHB before filling the sample chambers is adsorbed by the particles. Yet, also in this case, the degree of CHB dissociation must be very small, since the dispersion contains only few particles that are able to adsorb the ions. Another source of particle charges could be the protonation or deprotonation of amino functions and/or carboxylic acid groups on the particles, respectively. The particles used were synthesized from methylmetacrylate (MMA) containing roughly 2% of methacrylic acid (MA). MA is needed to allow for the covalent locking of the steric stabilizer on the particles. Thus, particles might become negatively charged by carboxylic acid deprotonation when in contact with surfaces such as glass during the filling procedure. van der Linde et al.^19^ report that they find higher charges on locked particles and explain this by the esterification of MA with the hydroxyl containing ring opening catalyst dimethylaminoethanol (DMAE) used for the locking. The amine function in DMAE could be charged positively by protonation. We can exclude this exact mechanism in our case, since we used dimethyldodecylamine as ring opening catalyst. Finally, the steric stabilizer itself also contains carboxylic acid groups. Charge on the particles would then be redistributed in the dispersion over the course of several days before reaching an equilibrium. The values for the various parameters that we determine after this equilibration process coincide well with earlier measurements of PMMA particles in CHB/*cis*-decalin. Using microelectrophoresis, van der Linden and co-workers were able to determine apparent surface charges between 456 and 1015 elementary charges for slightly smaller particles with a diameter of 1.98 *μ m*.^19^ Also there, strong variations in parameters like the apparent surface charge were reported in different samples despite identical preparation protocols. It is not known whether also in their case differences in the sample preparation times account for this behavior. One should note, however, that the authors assumed a Debye length of κ^–1^ = 6 *μ m*, significantly larger than our and other previously reported values for the same system.^23,48^

#### Influence of TBAB Addition on Interparticle Forces

As has been pointed out above, a common procedure to reach hard potentials in colloidal dispersions is to screen particle charges by the addition of salt. In organic dispersion media such as decalin used here, often TBAC or TBAB are employed.^11^ We therefore also investigated the effect of TBAB on the forces between PMMA dispersed in CHB/decalin.

**Figure 5:**
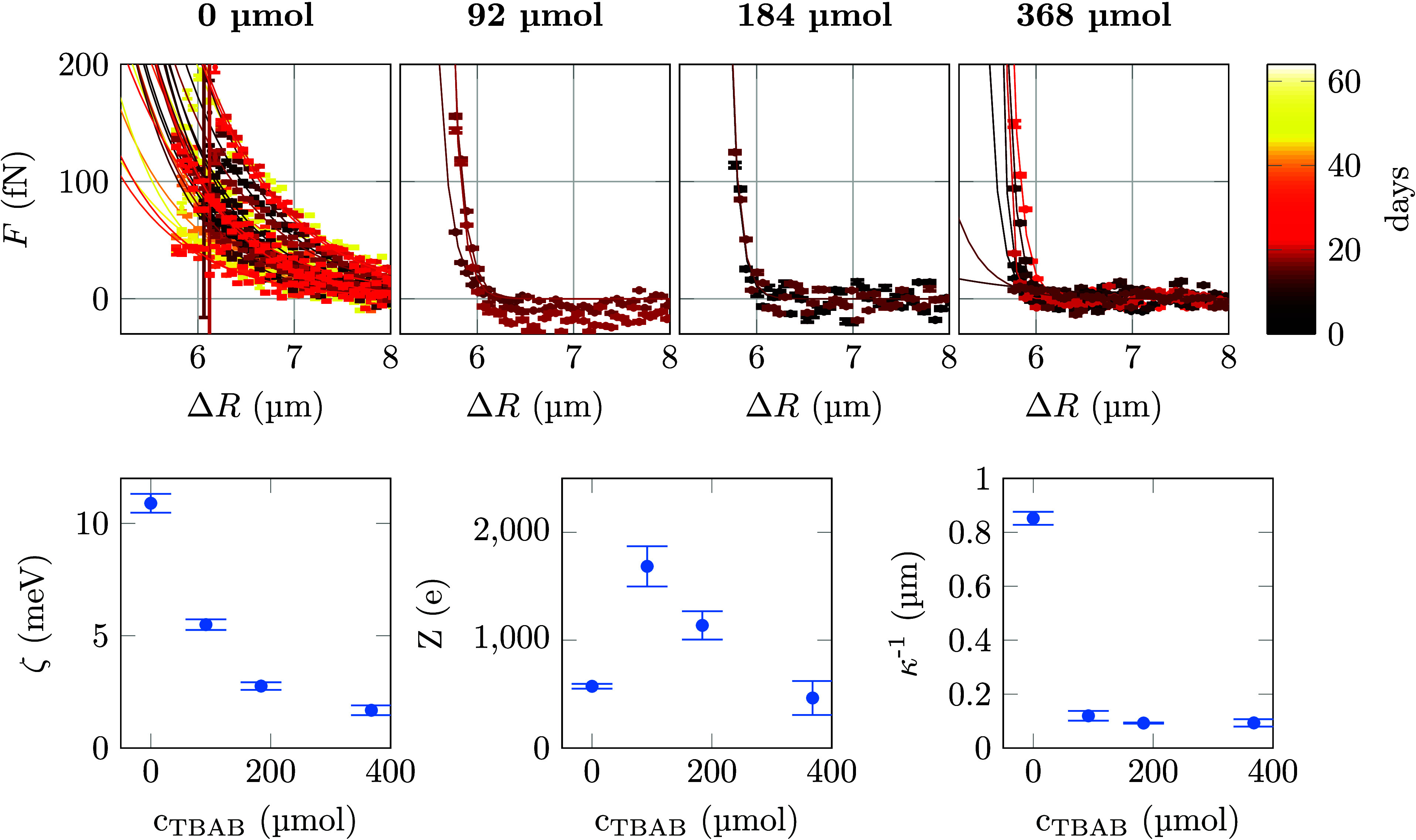
Upper row: Interparticle forces as a function of particle separation ΔR for different concentration of TBAB. The solid lines are fits to the data according to a screened Coulomb force (eq 1). Lower row: Data for the parameters collected in Table 1 plotted as a function of TBAB concentration. Error bars shown are 1 σ confidence intervals.

Measurements for four different TBAB concentrations *c*_*TBAB*_ (0 μmol, 92 μmol, 184 μmol and 368 μmol) are shown in Figure 5. The observed scattering of the data is due to the steepness of the potentials upon addition of TBAB. While, as stated above, each measurement is based on roughly 3 · 10^4^ individual image recordings, the respective distance dependent occurrences are distributed over the bins shown in the histogram of Figure 2 a. For the steep potentials observed upon addition of TBAB, most of the bins at larger distances are not relevant for the fitting. This leads to comparatively large errors of the fitting results. As discussed above, samples without added TBAB needed roughly 5 days to show a stable behavior, therefore only measurements that were taken more than 1 week after filling are shown in Figure 5. This allows a comparison of the influence of TBAB on the particle properties after charge redistribution. For each concentration, data for several colloid pairs were collected and each measurement shown is from a different particle pair. In the same sample cell, small differences between particle pairs are commonly observed. While we find a certain variation of the onset of the measured force increases that we attribute to the polydispersity of the particles, the slopes of the increases are very similar. This demonstrates a reasonable reproducibility of our data with added TBAB that has been reported to be difficult in earlier studies.^26^ The remaining differences between measurements might be due to the previously observed phenomenon of fluctuating charges on colloids.^49−51^ In addition, variations in particle charges could also result from different degrees of the covalent binding of the steric stabilizer, that is known to strongly influence the surface charge.^19^

None of the measured force curves show signs of an attractive potential. This is expected due to the fact that first, the steric stabilization of the PMMA particles reduces van der Waals forces and second, because our spatial resolution of 50 nm in Δ*R* is too small to observe the latter. The expected screening effect when adding TBAB is clearly visible from the shape of the force curves. Again, we determine values for the Debye lengths κ^–1^, for ζ potentials, and for apparent surface charges Z as described above. These values are collected in Table 1.

**Table 1:** Average Fitting Parameters from the Fits Shown in Figure 5^a^

*c*_*TBAB*_ (μmol)	*Nr*_*m*_	ζ (mV)	*Z* (*e*)	κ^–1^ (μm)	ϕ_*contact*_ (*k*_*B*_ *T*)
0	28	10.9 ± 0.4	572 ± 22	0.9 ± 0.03	106 ± 1.0
92	3	5.5 ± 0.2	1685 ± 186	0.1 ± 0.01	15 ± 1.4
184	2	2.8 ± 0.2	1137 ± 131	0.1 ± 0.01	4 ± 0.6
368	7	1.7 ± 0.2	464 ± 158	0.1 ± 0.01	1 ± 0.3

a Uncertainties are standard deviations. All fits and also Z are calculated with a value for the particle radius a determined by light microscopy. *Nr*_*m*_ is the number of measurements for a given TBAB concentration. ϕ_*contact*_ is the mean potential at contact, which was calculated by integration of eq 1 and inserting a as determined by light microscopy.

The determined κ^–1^ values indicate a clear change in the Debye length already when adding small concentrations of TBAB. Remarkably, the Debye length is roughly the same for all TBAB concentrations different from zero. This leads us to the conclusion, that above a concentration of 92 μmol TBAB, the free charge concentration in the sample liquid does not change by more than a factor of 2 anymore, as the Debye length directly correlates with the number of free charges in the dispersion fluid. For *c*_*TBAB*_ = 0 μmol, the value of κ^–1^ = 0.9 μm is in the same range as the value of 1.4 μm for dilute systems found by Royall and co-workers,^23^ but differs from κ^–1^ = 12 μm published earlier.^11^ The results also agree well with the findings of ref (52), where κ^–1^ ≈ 1.2 μm was determined for *c*_*TBAB*_ = 0 μmol and κ^–1^ ≈ 0.2 μm for *c*_*TBAB*_ > 50 μmol in a system of 2.8 μm sized PMMA particles in CHB/cis-decalin. Similarly Leunissen and colleagues found κ^–1^ = 195 nm for *c*_*TBAB*_ = 190 μmol.^48^

As pointed out, it is not possible for us to determine the sign of ζ. Previous electrophoretic mobility measurements showed a change from positive to negative ζ potentials of the particles, when the TBAB concentration was added.^12,27,53^ We therefore assume that also in our case, the particle’s ζ potentials changed from being positive without added TBAB to being negative for higher concentrations of TBAB. This means that we do not expect to observe a monotonous decrease of ζ as observed by Kim et al.^27^ The absolute average charge *Z* has its maximum value of 1685 elementary charges at *c*_*TBAB*_ = 92 μmol. Since the apparent surface charge *Z* is itself linearly dependent on ζ, the sign of which cannot be determined with our method, also the sign of ζ remains unknown. Our values for Z compare well with experimental data reported earlier such as *Z* = +221 *e* in CHB/decalin (27 wt %) for 2.8 μm PMMA spheres.^52^

From the data depicted in Figure 5 it is obvious that the effects from the addition of TBAB are so strong that in this case and in contrast to the samples made from pure CHB/decalin, no temporal evolution of the particle interaction is detected. This can be rationalized by the large number of ions present in the dispersion fluid after TBAB addition that overwhelms any small changes due to charge exchange between particles and fluid.

## Conclusion

In this work, we report measurements of forces between individual pairs of PMMA particles dispersed in decalin. To measure these forces directly, we employed a variant of the blinking tweezers method.^31^ The forces that we determine agree well with previously published data. As has been reported before by van der Linden and co-workers,^19^ we also find, however, that the observed interparticle forces varied strongly. Using measurement series extending over tens of days, we could show that the variations are not due to impurities introduced during sample preparation, but to the temporal evolution of the particle charging. Since we found no concomitant temporal change of the Debye lengths, we assume that the charging of the particles is not caused by dissociation of CHB, but by the filling procedure. Quantitative measurements of the effect of the addition of TBAB to the dispersion as a screening agent shows that this increased the hardness of the particle potential. Already small amounts of TBAB lead to a large effect. In this case no temporal change of the interparticle forces was detected. However, even after TBAB addition, the particle potentials are not perfectly hard, as the contact potential even at the highest TBAB concentration was found to be approximately 1 *k*_*B*_*T*. Depending on the application, it might thus be necessary to find alternatives to the system described here. Recent investigations by Kale and co-workers show that dispersions of sterically stabilized PMMA particles in mixtures of decalin with tetrachloroehtylene show hard sphere behavior,^26^ albeit at the price of a high toxicity of the dispersion medium.

## References

[ref1] Manoharan V. N. Colloidal matter: Packing, geometry, and entropy. Science 2015, 349, 1253751 10.1126/science.1253751. 26315444

[ref2] van Blaaderen A.; Wiltzius P. Real-Space Structure of Colloidal Hard-Sphere Glasses. Science 1995, 270, 1177–1179. 10.1126/science.270.5239.1177.

[ref3] Crocker J. C.; Grier D. G. Methods of digital video microscopy for colloidal studies. J. Colloid Interface Sci. 1996, 179, 298–310. 10.1006/jcis.1996.0217.

[ref4] Poon W. Colloids as big atoms. Science 2004, 304, 830–831. 10.1126/science.1097964. 15131294

[ref5] Weeks E. R.; Crocker J. C.; Levitt A. C.; Schofield A.; Weitz D. A. Three-dimensional direct imaging of structural relaxation near the colloidal glass transition. Science 2000, 287, 627–631. 10.1126/science.287.5453.627. 10649991

[ref6] Weeks E. R. Introduction to the Colloidal Glass Transition. ACS Macro Lett. 2017, 6, 27–34. 10.1021/acsmacrolett.6b00826. 35632870

[ref7] Wiederseiner S.; Andreini N.; Epely-Chauvin G.; Ancey C. Refractive-index and density matching in concentrated particle suspensions: a review. Exp. Fluids 2011, 50, 1183–1206. 10.1007/s00348-010-0996-8.

[ref8] Kodger T.; Guerra R. E.; Sprakel J. Precise colloids with tunable interactions for confocal microscopy. Sci. Rep. 2015, 5, 14635–14645. 10.1038/srep14635. 26420044 PMC4588590

[ref9] Park N.; Umanzor E. J.; Conrad J. C. Aqueous Colloid and Polymer Depletion System for Confocal Microscopy and Rheology. Front. Phys. 2018, 6, 42 10.3389/fphy.2018.00042.

[ref10] Liu Y.; Yanagishima T.; Curran A.; Edmond K. V.; Sacanna S.; Dullens R. P. A. Colloidal Organosilica Spheres for Three-Dimensional Confocal Microscopy. Langmuir 2019, 35, 7962–7969. 10.1021/acs.langmuir.9b00963. 31095907

[ref11] Yethiraj A.; van Blaaderen A. A colloidal model system with an interaction tunable from hard sphere to soft and dipolar. Nature 2003, 421, 513–517. 10.1038/nature01328. 12556887

[ref12] Royall C. P.; Leunissen M. E.; van Blaaderen A. A new colloidal model system to study long-range interactions quantitatively in real space. J. Phys.: Cond. Matt. 2003, 15, S3581–S3596. 10.1088/0953-8984/15/48/017.

[ref13] Kaufman L. J.; Weitz D. A. Direct imaging of repulsive and attractive colloidal glasses. J. Chem. Phys. 2006, 125, 074716 10.1063/1.2227386. 16942373

[ref14] Edmond K. V.; Elsesser M. T.; Hunter G. L.; Pine D. J.; Weeks E. R. Decoupling of rotational and translational diffusion in supercooled colloidal fluids. Proc. Natl. Acad. Sci. U.S.A. 2012, 109, 17891–17896. 10.1073/pnas.1203328109. 23071311 PMC3497755

[ref15] Besseling T. H.; Hermes M.; Fortini A.; Dijkstra M.; Imhof A.; van Blaaderen A. Oscillatory shear-induced 3D crystalline order in colloidal hard-sphere fluids. Soft Matter 2012, 8, 6931–6939. 10.1039/c2sm07156h.

[ref16] Wood N.; Russo J.; Turci F.; Royall C. P. Coupling of sedimentation and liquid structure: Influence on hard sphere nucleation. J. Chem. Phys. 2018, 149, 204506 10.1063/1.5050397. 30501259

[ref17] Roller J.; Laganapan A.; Meijer J.-M.; Fuchs M.; Zumbusch A. Observation of liquid glass in suspensions of ellipsoidal colloids. Proc. Natl. Acad. Sci. U.S.A. 2021, 118, e2018072118 10.1073/pnas.2018072118. 33397813 PMC7826331

[ref18] Royall C. P.; Poon W. C. K.; Weeks E. R. In search of colloidal hard spheres. Soft Matter 2013, 9, 17–27. 10.1039/C2SM26245B.

[ref19] van der Linden M. N.; Stiefelhagen J. C. P.; Heessels-Gürboğa G.; van der Hoeven J. E. S.; Elbers N. A.; Dijkstra M.; van Blaaderen A. Charging of poly(methyl methacrylate) (PMMA) colloids in cyclohexyl bromide: locking, size dependence, and particle mixtures. Langmuir 2015, 31, 65–75. 10.1021/la503665e. 25535669

[ref20] Choi K. H.; Kang D. W.; Kim K. H.; Kim J.; Lee Y.; Im S. H.; Park B. J. Direct measurement of electrostatic interactions between poly(methyl methacrylate) microspheres with optical laser tweezers. Soft Matter 2019, 15, 8051–8058. 10.1039/C9SM01374A. 31549697

[ref21] Bryant G.; Williams S. R.; Qian L.; Snook I. K.; Perez E.; Pincet F. How hard is a colloidal “hard-sphere” interaction?. Phys. Rev. E 2002, 66, 060501 10.1103/PhysRevE.66.060501. 12513261

[ref22] Hsu M. F.; Dufresne E. R.; Weitz D. A. Charge Stabilization in Nonpolar Solvents. Langmuir 2005, 21, 4881–4887. 10.1021/la046751m. 15896027

[ref23] Royall C. P.; Leunissen M. E.; Hynninen A.-P.; Dijkstra M.; van Blaaderen A. Re-entrant melting and freezing in a model system of charged colloids. J. Chem. Phys. 2006, 124, 244706 10.1063/1.2189850. 16821995

[ref24] Espinosa C. E.; Guo Q.; Singh V.; Behrens S. H. Particle Charging and Charge Screening in Nonpolar Dispersions with Nonionic Surfactants. Langmuir 2010, 26, 16941–16948. 10.1021/la1033965. 20942432

[ref25] Iacovella C. R.; Rogers R. E.; Glotzer S. C.; Solomon M. J. Pair interaction potentials of colloids by extrapolation of confocal microscopy measurements of collective suspension structure. J. Chem. Phys. 2010, 133, 164903 10.1063/1.3498746. 21033819

[ref26] Kale S.; Lederer A.; Oettel M.; Schöpe H. J. Approaching the hard sphere limit in colloids suitable for confocal microscopy– the end of a decade lasting quest. Soft Matter 2023, 19, 2146–2157. 10.1039/D2SM01427K. 36880153

[ref27] Kim Y.; Shah A. A.; Solomon M. Spatially and temporally reconfigurable assembly of colloidal crystals. Nat. Commun. 2014, 5, 3676 10.1038/ncomms4676. 24759549

[ref28] Farrokhbin M.; Stojimirović B.; Galli M.; Khajeh Aminian M.; Hallez Y.; Trefalt G. Surfactant mediated particle aggregation in nonpolar solvents. Phys. Chem. Chem. Phys. 2019, 21, 18866–18876. 10.1039/C9CP01985E. 31436779

[ref29] Pusey P. N.; van Megen W. Phase behaviour of concentrated suspensions of nearly hard colloidal spheres. Nature 1986, 320, 340–342. 10.1038/320340a0.

[ref30] Dinsmore A. D.; Weeks E. R.; Prasad V.; Levitt A. C.; Weitz D. A. Three-dimensional confocal microscopy of colloids. Appl. Opt. 2001, 40, 4152–4159. 10.1364/AO.40.004152. 18360451

[ref31] Sainis S. K.; Germain V.; Dufresne E. R. Statistics of particle trajectories at short time intervals reveal fN-scale colloidal forces. Phys. Rev. Lett. 2007, 99, 018303 10.1103/PhysRevLett.99.018303. 17678194

[ref32] Sainis S. K.; Germain V.; Mejean C. O.; Dufresne E. R. Electrostatic Interactions of Colloidal Particles in Nonpolar Solvents: Role of Surface Chemistry and Charge Control Agents. Langmuir 2008, 24, 1160–1164. 10.1021/la702432u. 18062711

[ref33] Sainis S. K.; Germain V.; Mejean C. O.; Dufresne E. R. Electrostatic Interactions of Colloidal Particles in Nonpolar Solvents: Role of Surface Chemistry and Charge Control Agents. Langmuir 2008, 24, 1160–1164. 10.1021/la702432u. 18062711

[ref34] Gutsche C.; Elmahdy M. M.; Kegler K.; Semenov I.; Stangner T.; Otto O.; Ueberschär O.; Keyser U. F.; Krueger M.; Rauscher M.; Weeber R.; Harting J.; Kim Y. W.; Lobaskin V.; Netz R. R.; Kremer F. Micro-rheology on (polymer-grafted) colloids using optical tweezers. J. Phys.: Cond. Matt. 2011, 23, 184114 10.1088/0953-8984/23/18/184114. 21508470

[ref35] El Masri D.; van Oostrum P.; Smallenburg F.; Vissers T.; Imhof A.; Dijkstra M.; van Blaaderen A. Measuring colloidal forces from particle position deviations inside an optical trap. Soft Matter 2011, 7, 3462–3466. 10.1039/c0sm01295e.

[ref36] Evans D. J.; Hollingsworth A. D.; Grier D. G. Charge renormalization in nominally apolar colloidal dispersions. Phys. Rev. E 2016, 93, 042612 10.1103/PhysRevE.93.042612. 27176357

[ref37] Crocker J. C.; Grier D. G. Microscopic measurement of the pair interaction potential of charge-stabilized colloid. Phys. Rev. Lett. 1994, 73, 352–355. 10.1103/PhysRevLett.73.352. 10057148

[ref38] Klein M. K.; Saenger N. R.; Schuetter S.; Pfleiderer P.; Zumbusch A. Shape-Tunable Core–Shell Microparticles. Langmuir 2014, 30, 12457–12464. 10.1021/la500504u. 24649803

[ref39] Wu Y. L.; Derks D.; van Blaaderen A.; Imhof A. Melting and crystallization of colloidal hard-sphere suspensions under shear. Proc. Natl. Acad. Sci. U.S.A. 2009, 106, 10564–10569. 10.1073/pnas.0812519106. 19541643 PMC2705612

[ref40] Everts J. C.; Samin S.; Elbers N. A.; van der Hoeven J. E. S.; Van Blaaderen A.; van Roij R. Colloid-oil-water-interface interactions in the presence of multiple salts: charge regulation and dynamics. Phys. Chem. Chem. Phys. 2017, 19, 14345–14357. 10.1039/C7CP01935A. 28537607

[ref41] Roller J.; Geiger J. D.; Voggenreiter M.; Meijer J.-M.; Zumbusch A. Formation of nematic order in 3D systems of hard colloidal ellipsoids. Soft Matter 2020, 16, 1021–1028. 10.1039/C9SM01926J. 31854439

[ref42] El Masri D.; Vissers T.; Badaire S.; Stiefelhagen J. C. P.; Vutukuri H. R.; Helfferich P.; Zhang T. H.; Kegel W. K.; Imhof A.; van Blaaderen A. A qualitative confocal microscopy study on a range of colloidal processes by simulating microgravity conditions through slow rotations. Soft Matter 2012, 8, 6979–6990. 10.1039/c2sm07217c.

[ref43] Zargar R.; Nienhuis B.; Schall P.; Bonn D. Direct Measurement of the Free Energy of Aging Hard Sphere Colloidal Glasses. Phys. Rev. Lett. 2013, 110, 258301 10.1103/PhysRevLett.110.258301. 23829762

[ref44] Elsesser M. T.; Hollingsworth A. D. Revisiting the Synthesis of a Well-Known Comb-Graft Copolymer Stabilizer and Its Application to the Dispersion Polymerization of Poly(methyl methacrylate) in Organic Media. Langmuir 2010, 26, 17989–17996. 10.1021/la1034917. 21053983

[ref45] Schütter S.; Roller J.; Kick A.; Meijer J.-M.; Zumbusch A. Real-space imaging of translational and rotational dynamics of hard spheres from the fluid to the crystal. Soft Matter 2017, 13, 8240–8249. 10.1039/C7SM01400G. 29063943

[ref46] Green J. H. S.; Maccoll A. Studies in the pyrolysis of organic bromides. Part V. The pyrolysis of cyclohexyl bromide. J. Chem. Soc. 1955, 2449–2454. 10.1039/jr9550002449.

[ref47] Golinkin H. S.; Parbhoo D. M.; Robertson R. E. Solvation differences in the hydrolysis of certain alicyclic bromides. Can. J. Chem. 1970, 48, 1296–1301. 10.1139/v70-213.

[ref48] Leunissen M. E.; Christova C. G.; Hynninen A.-P.; Royall C. P.; Campbell A. I.; Imhof A.; Dijkstra M.; van Roij R.; van Blaaderen A. Ionic colloidal crystals of oppositely charged particles. Nature 2005, 437, 235 10.1038/nature03946. 16148929

[ref49] Strubbe F.; Beunis F.; Neyts K. Determination of the effective charge of individual colloidal particles. J. Colloid Interface Sci. 2006, 301, 302–309. 10.1016/j.jcis.2006.04.034. 16765364

[ref50] Strubbe F.; Beunis F.; Neyts K. Detection of Elementary Charges on Colloidal Particles. Phys. Rev. Lett. 2008, 100, 218301 10.1103/PhysRevLett.100.218301. 18518642

[ref51] Schreuer C.; Vandewiele S.; Brans T.; Strubbe F.; Neyts K.; Beunis F. Single charging events on colloidal particles in a nonpolar liquid with surfactant. J. Appl. Phys. 2018, 123, 015105 10.1063/1.5012887.

[ref52] Elbers N. A.; van der Hoeven J. E. S.; de Winter D. A. M.; Schneijdenberg C. T. W. M.; van der Linden M. N.; Filion L.; van Blaaderen A. Repulsive van der Waals forces enable Pickering emulsions with non-touching colloids. Soft Matter 2016, 12, 7265–7272. 10.1039/C6SM01294A. 27406917

[ref53] Leunissen M. E.; Zwanikken J.; van Roij R.; Chaikin P. M.; van Blaaderen A. Ion partitioning at the oil–water interface as a source of tunable electrostatic effects in emulsions with colloids. Phys. Chem. Chem. Phys. 2007, 9, 6405–6414. 10.1039/b711300e. 18060171

